# High Cycle Fatigue Performance of LPBF 304L Stainless Steel at Nominal and Optimized Parameters

**DOI:** 10.3390/ma13071591

**Published:** 2020-03-31

**Authors:** Mohammad Masud Parvez, Tan Pan, Yitao Chen, Sreekar Karnati, Joseph W. Newkirk, Frank Liou

**Affiliations:** 1 Department of Mechanical and Aerospace Engineering, Missouri University of Science and Technology, Rolla, MO 65401, USA; tpb44@umsystem.edu (T.P.); yc4gc@umsystem.edu (Y.C.); skw92@umsystem.edu (S.K.); liou@umsystem.edu (F.L.); 2 Material Science and Engineering, Missouri University of Science and Technology, Rolla, MO 65401, USA; jnewkirk@umsystem.edu

**Keywords:** additive manufacturing, 304L stainless steel, tensile test, impact toughness test, fatigue test, nucleation and propagation, miniature specimen, simply supported bending

## Abstract

In additive manufacturing, the variation of the fabrication process parameters influences the mechanical properties of a material such as tensile strength, impact toughness, hardness, fatigue strength, and so forth, but fatigue testing of metals fabricated with all different sets of process parameters is a very expensive and time-consuming process. Therefore, the nominal process parameters by means of minimum energy input were first identified for a dense part and then the optimized process parameters were determined based on the tensile and impact toughness test results obtained for 304L stainless steel deposited in laser powder bed fusion (LPBF) process. Later, the high cycle fatigue performance was investigated for the material built with these two sets of parameters at horizontal, vertical, and inclined orientation. In this paper, displacement controlled fully reversed (R = −1) bending type fatigue tests at different levels of displacement amplitude were performed on Krouse type miniature specimens. The test results were compared and analyzed by applying the control signal monitoring (CSM) method. The analysis shows that specimen built-in horizontal direction for optimized parameters demonstrates the highest fatigue strength while the vertical specimen built with nominal parameters exhibits the lowest strength.

## 1. Introduction

Additive manufacturing (AM) has recently attained much popularity both in research and application fields such as aerospace, automobile, maritime, biomedical, and other industrial sectors [1,2,3]. Among different additive manufacturing process available, laser powder bed fusion (LPBF) also known as selective laser melting (SLM) is a widely accepted method to fabricate metals and alloys [4,5,6,7,8]. LPBF is capable of depositing near-net-shape internal and external complex geometries but the major drawbacks of the AM materials are the surface irregularities, residual stress, and defects such as porosity, microcracks, inclusions, dislocations, and others. They significantly influence the static and dynamic mechanical properties of a material including fatigue strength. Several studies have been carried out recently to assess the fatigue behavior of different AM materials, that is, AlSi10Mg [9,10,11,12], Ti6Al4V [13,14,15,16,17,18], Ni-based alloy [19], 15-5 PH stainless steel [20], steel [21], stainless steel [22]. Nasab et al. [23] studied the effect of surface and subsurface defects on the fatigue behavior of AlSi10Mg. They also investigated the combined effect of surface anomalies and volumetric defects on fatigue life of AlSi7Mg fabricated via laser powder bed fusion [24]. Romano [25] investigated the effect of sub-surface porosity and surface roughness on the high cycle fatigue behavior of AM 17-4 PH stainless steel. Chan et al. [26] developed a methodology to predict the surface crack nucleation mechanism in a nickel-based superalloy AM 718Plus and proposed that fatigue life enhancement can be achieved by machining and polishing the surface. Zhan et al. [27] proposed a fatigue damage model considering AM effects and investigated the variation of fatigue life with the volumetric energy density and the variation of damage evolution rate. Biswal et al. [28] studied the effect of internal porosity on the fatigue strength of Ti-6Al-4V and proposed a modified Kitagawa-Takahashi diagram and a critical pore diameter to initiate crack applying Murakami’s approach. Since in AM, the formation of different types of porosity, that is, lack of fusion, keyhole, voids, and others is substantially influenced by the fabrication process parameters, the effect of process parameters on the fatigue behavior is yet to be investigated. While minimum energy input for a dense part originates lack of fusion type porosity most, keyhole defects are dominant at high energy input [29,30,31].

Due to the layer-by-layer deposition process in AM technique, another downside of the AM material is the anisotropy issue in mechanical behaviors that is, tensile performance [32,33,34,35,36,37,38], impact toughness [39] and fatigue properties [40]. Guan et al. [33] investigated the potential variables causing the anisotropy issue. They reported that both layer thickness and overlap rate showed an insignificant effect on the tensile properties on account of the similar metallurgical bonding and microstructure while build direction and hatch angle revealed strong impacts on mechanical properties by influencing stress concentration and microstructure. Hatch angle is the laser scan path angle with the x-axis. Wang et al. [34] and Yu et al. [37] further confirmed the anisotropy in tensile properties with different build orientations and proposed that columnar grain structure with higher length-width ratio induced by the rapid cooling rate of AM process could be the main reason for the mechanical anisotropies. Karnati et al. [39] investigated the anisotropy issue in impact toughness of AM printed AISI 304L stainless steel with different build orientations where vertical Charpy specimens exhibited the lowest toughness while horizontal specimens provided the highest. They explained the difficulty level of crack propagation along the interlayer track boundaries could be the possible reason. While Blinn et al. [40] studied the anisotropic fatigue behavior of AISI 316L stainless steel, the effect of anisotropy on the nucleation and propagation phase in fatigue assessment is yet to be investigated.

In this paper, the effect of build process parameters and anisotropy issues on the fatigue behavior of laser powder bed fused AISI 304L stainless steel was investigated. For fatigue testing, two sets of process parameters were selected to fabricate parts at horizontal, vertical, and inclined build direction. Nominal parameters were chosen for minimum energy input for a dense part while the optimized parameters were determined by performing tensile and impact toughness tests. The fatigue test was conducted on miniature specimens. Since fabricating standard specimen is very expensive and time-consuming in the AM process, miniature specimens are recently employed in mechanical properties characterization of an AM material. Sreekar et al. [41] showed the miniature specimens to be instrumental in characterizing both bulk and AM material properties reliably. Dzugan et al. [42] demonstrated the implementation of miniaturized tensile test specimens instead of standard specimens in determining the local properties characterization of AM material Ti-6Al-4V. Wan et al. [43] addressed the reasons, necessities, and potential strategies to evaluate and qualify the fatigue performance of AM material using miniature specimens.

A modified Krouse type specimen with a dual gauge section was implemented for the bending fatigue test in this paper. The Krouse type specimen is a wedge-shaped ASTM (American Society for Testing and Materials) International standard B593-96(2014)e1 specimen, definition E206, and practice E468 [44]. The advantage of using such specimens is that nominal stress distribution remains uniform within the gauge. Haidyrah et al. [45,46] and Gohil et al. [47] applied finite element analysis method on different modified versions of Krouse type specimens to confirm the uniform nominal stress distribution within the gauge. The uniform stress distribution eliminates the stress gradient effect in the bending type test of miniature specimens [48,49]. Additionally, the increased surface area in a dual gauge specimen may also capture different surface and subsurface defects. A displacement controlled test was performed in this study since the displacement controlled fatigue test tends to be a more stable approach than load control and the driving force decreases with the crack growth in displacement control [50]. Later implementing the control signal monitoring (CSM) method [51,52], the crack nucleation, and propagation phase were identified and compared for different build directions and process parameters.

## 2. Materials and Methodology

### 2.1. Materials

The material used for this study was Ar gas atomized AISI 304L stainless steel (SS) powder purchased from LPW Technology (Imperial, PA 15126, USA) with a reported particle size distribution ranging from 15 to 45 μm and true powder density of 7.935 g/cm^3^. The chemical composition provided by the vendor is listed in Table 1. From the chemistry of the material, this may be characterized as 304L SS due to its low content of interstitial, N, C, O, and so forth.

### 2.2. Fabrication

The AM machine used in this study was Renishaw AM 250 (Renishaw Inc., Auburn Hills, MI 48326, USA) equipped with an Nd-YAG pulsed laser (IPG Photonics, Oxford, MA 01540, USA). The maximum power capacity of the laser is 200 W with a Gaussian intensity profile. A preheating process was employed prior to the initiation of the build. The substrate and powder were heated up and maintained 80 °C temperature to reduce the thermal gradient and eliminate the water vapor inside powder particles. During the printing process, a recirculating Ar gas flow was maintained to remove the condensate generated. A design of experiment (DOE) was implemented for the selection of process parameters in this study in order to vary the energy density range. The energy density (ED) in AM is defined as

(1) $$\begin{array}{cc} ED=\frac{P}{v\times h\times t} & (J/mm^{3}), \end{array}$$

where, *P*, *v*, *h*, and *t* are the laser power (W), scan speed (m/s), hatch spacing (μm), and layer thickness (μm), respectively. Hatch spacing is defined as the distance between two adjacent laser scan tracks. Scan speed was calculated using Equation (2)

(2) $$\begin{array}{cc} v=\frac{\;\mathrm{Point}\;\mathrm{distance}}{\mathrm{Exposure}\;\mathrm{time}} & (m/s). \end{array}$$

Point distance and hatch spacing were identified as two variables where the scan speed of the laser traveling was linearly related to the point distance. A higher point distance determines a higher scan speed, hence a lower energy input is achieved and vice versa. Hatch spacing controls the overlap between adjacent laser track, which tends to cause the lack of fusion defects with less overlap and keyhole porosity with higher overlay. A previous study by Brown [53] proposed a nominal parameter for AISI 304L which originated from the optimization of bulk density and surface finish of downward skin. The parameters were selected in a range surrounding the nominal parameters. Three levels of point distance and five levels of hatch spacing were designated to obtain a 2-way full factorial experiment with 15 parameter combinations. The laser power was set at 200 W with a constant layer thickness of 50 μm and an exposure time of 88 μs. A stripe scan pattern was applied to guide the way laser scanned with a hatch angle of 67° rotating between two adjacent layers. The 15 parameter combinations are listed in Table 2 where ED #8 is for the nominal parameters mentioned here [53].

### 2.3. Parameters Selection

#### 2.3.1. Nominal Parameters

**Figure 1:**
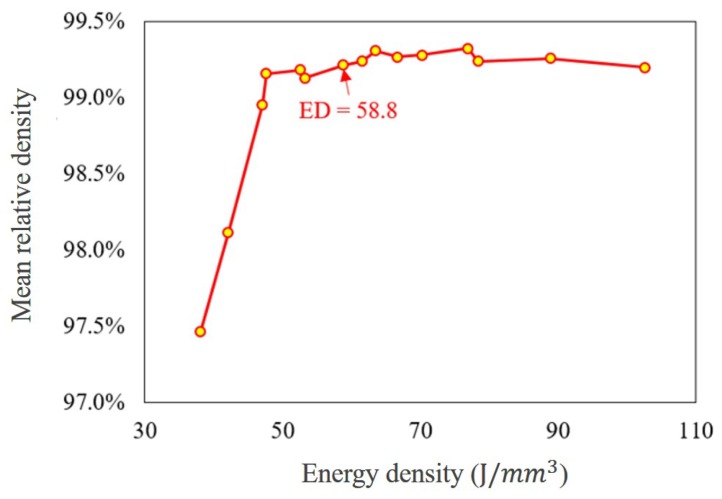
Relative density of the samples printed with the combination of all parameters.

In order to determine the nominal parameters, a density test was performed after the samples were printed with parameters listed in Table 2. All the samples were cut off from the substrate with a Sodick VZ300L Wire Electric Discharge Machine (EDM). The density test was performed on the as-printed samples based on the Archimedes principle. The mean relative density results are illustrated in Figure 1 with a true powder density of 7.935 g/cm^3^ and the average of three samples. From the trend of the relative volumetric density along with the energy density indicated at the lower ED region, the relative density of the as-printed sample elevated with the increase of ED till 58.8 J/mm^3^. After that, the relative density was almost consistent (>99.0%) with negligible fluctuation. Therefore, the set of parameters for ED #8 was chosen as the nominal parameters in this study [53].

#### 2.3.2. Optimized Parameters

**Figure 2:**
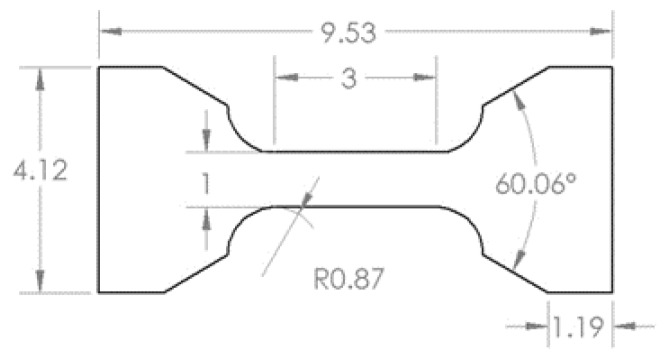
Dimension of the miniature tensile specimen. Units are in mm.

**Figure 3:**
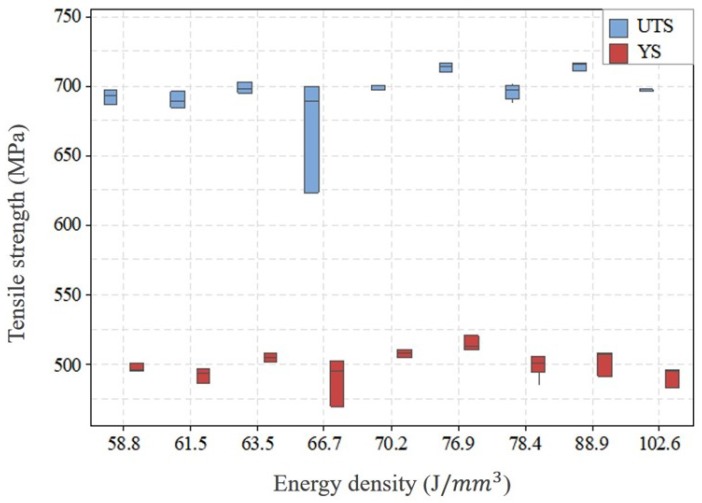
Tensile strength (yield strength (YS) and ultimate tensile strength (UTS)) of horizontal specimens with different parameters combination.

**Figure 4:**
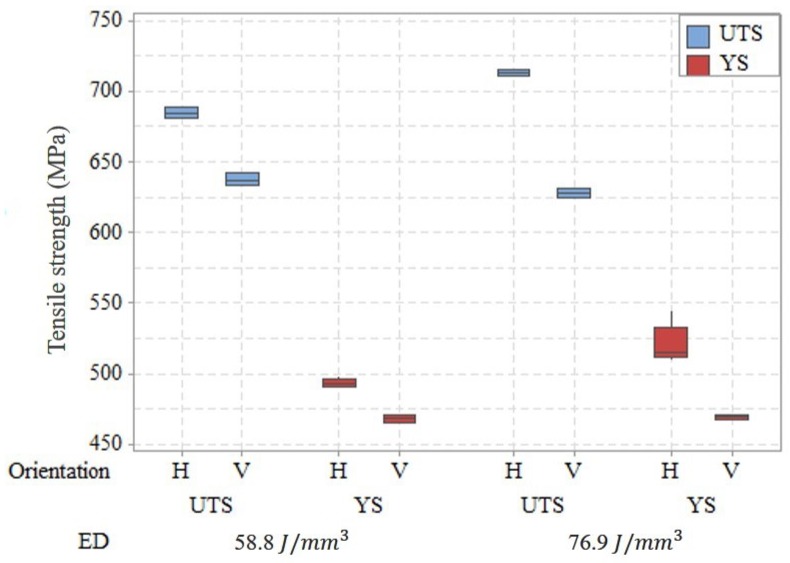
Tensile strength (YS and UTS) of horizontal and vertical specimens for energy densities 58.8 and 76.9 J/mm^3^.

**Figure 5:**
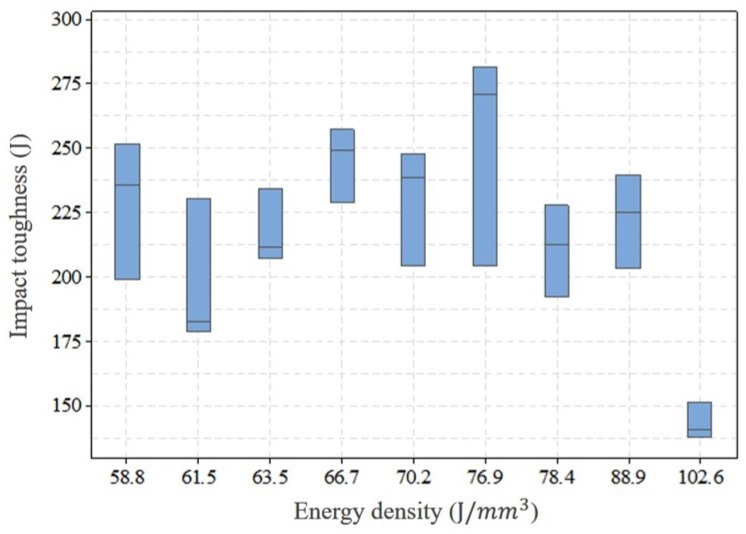
The impact toughness of as-built Charpy specimens printed in the vertical orientation with different parameters combination.

In order to determine the optimized parameters, tensile and impact toughness test was performed on the samples with ED above 58.8 J/mm^3^ included. Tensile testing was performed with miniature dog bone specimen as shown in Figure 2 [41]. The samples fabricated were machined to size with a nominal thickness of 1 mm. Three specimens were prepared for each parameter combination with the gauge length perpendicular to the build direction (horizontal specimen). Prior to the testing, the specimens were polished with 800 grit sandpaper. The Instron UTM machine was employed in this study to do tensile testing. The strain was controlled using an extensometer with a strain rate of 0.015 mm/min to 1% strain value. After that, the extensometer was removed and the testing was run with a cross-head speed of 1.5 mm/min. The tensile properties of the tested samples were represented by 0.2% offset yield strength (YS) and ultimate tensile strength (UTS). From the tensile test result of horizontal specimens with different energy density combinations as shown in Figure 3, it can be seen that no significant differences in YS and UTS can be recognized within the current ED range though there is a large scatter in results at an energy density of 66.7 J/mm^3^. The possible reason could be the quality of the printed part affected by the location on the build plate and the relative location of the gas flow [36]. However, the median of YS and UTS at horizontal orientation reached the maximum value at 76.9 J/mm^3^ in comparison with other parameter combinations while the difference in UTS and YS for the vertical orientation (shown in Figure 4) at 58.8 and 76.9 J/mm^3^ is negligible. Therefore, a further check was performed on vertical orientation by Charpy testing to the as-printed sample to demonstrate the parameter set optimized for impact toughness according to ASTM Standard E23. The longest axis of the Charpy specimen was printed along the build direction. A 2 mm “V” notch was machined with a standard broach. Three specimens were tested at each ED. The result of impact toughness from Charpy testing is illustrated in Figure 5. A relatively larger variation of impact toughness was observed for all ED. The median toughness achieved the optimal value at 76.9 J/mm^3^. Hence, 76.9 J/mm^3^ was demonstrated to be an optimized parameter set for both tensile strength and toughness.

## 3. Fatigue Test

### 3.1. Specimen Design and Preparation

The fatigue strength depends on the specimen size, dimension, and loading condition. Small specimens demonstrate higher fatigue strength compared to standard specimens while real parts exhibit even lower strength than the standard ones. While extended studies have been carried out to compare and evaluate the size effects on fatigue tests [54,55,56,57,58,59,60,61,62,63,64,65,66,67,68], recent studies show the implementation of miniature specimens to be instrumental in characterizing local properties of AM materials [41,42,43].

**Figure 6:**
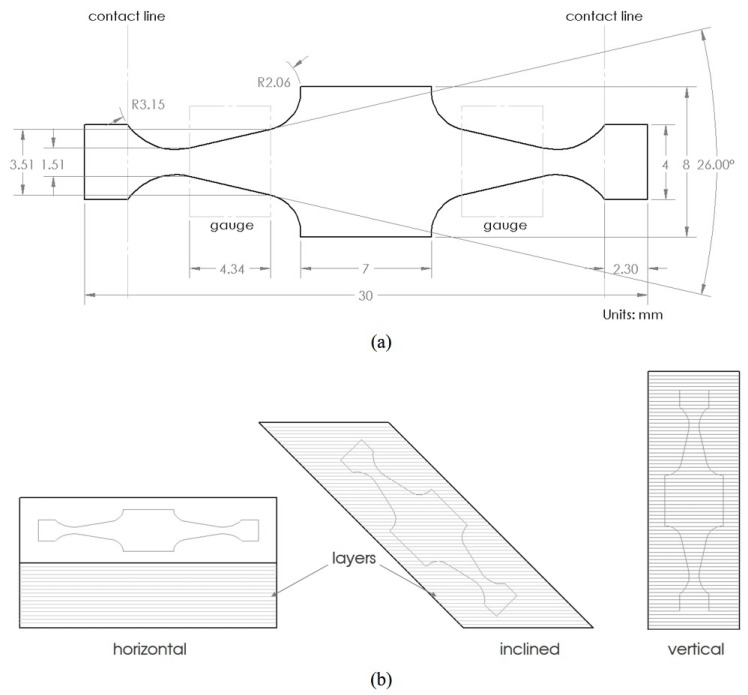
Schematic representation of (**a**): the miniature specimen including the dimensions with the dual gauge section and line contact, (**b**): the specimen cut at horizontal, inclined, and vertical orientation [52].

Since in AM, the effect of the process and build orientation could lead to fatigue property differences within the same part, therefore, a miniature specimen was designed. The dimension of the specimen is shown in Figure 6. The nominal thickness of the specimen was 0.67 mm. The Krouse type specimen with a dual gauge section was employed to conduct fatigue testing on the specimen fabricated using nominal and optimized parameters. While the Krouse type specimen ensures uniform stress distribution, the dual gauge with increased surface area enhances the probability of capturing different kinds of surface and subsurface defects since fatigue crack initiates at this location in most cases. The dual gauge also helps in distributing the load symmetrically and keeping the actuation path unidirectional, unlike a single cantilever beam. The specimen was specially designed for simply-supported test conditions. One of the advantages of using simply-supported is that the displacement is four times the displacement in a fully clamped mechanism. Since fatigue strength is also greatly influenced by the surface roughness of the test sample, therefore, finely finished (average roughness $R_{a}=0.482$ μm, average $R_{z}=4.242$ μm) specimens were machined using a W-EDM. A total of 7 specimens for each build orientation and process parameters were prepared without any additional surface preparation. Detailed FEA simulation results and sensitivity analysis on the designed specimen can be found here [51].

### 3.2. Test Setup

**Figure 7:**
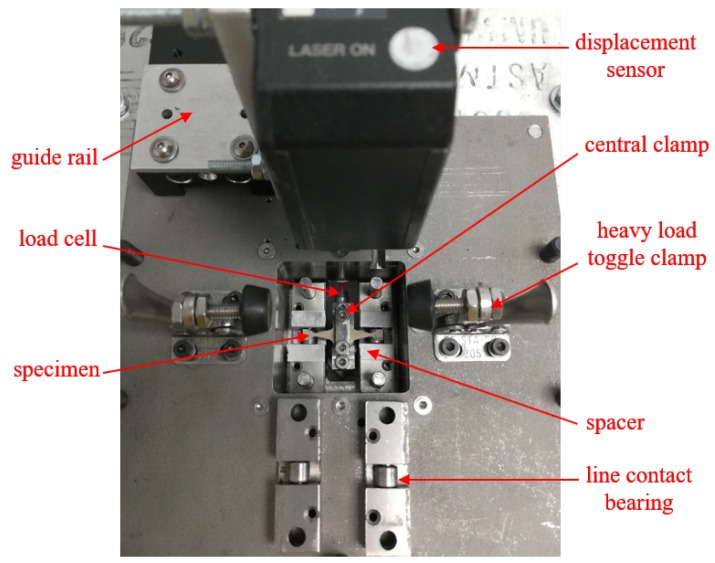
Fatigue test bench setup [51].

An additional advantage of using miniature specimens is that it requires a lower range of force; thus it minimizes the test setup cost. A subwoofer with low power capacity was employed as an actuator in this study. The subwoofer is a low-frequency drive. The mechanical modeling of a subwoofer is relatively similar to an electrodynamic shaker but the shaker has more rigid moving parts. Since higher rigidity requires higher force, the subwoofer with soft mechanical suspension is a well fitted low-cost alternate of a shaker. In order to utilize the woofer as the actuator, a plastic flange of 76.2 mm diameter replaced the cap of the voice coil. The flange of relatively larger diameter (3 times) compared to the effective length of the specimen (25.4 mm) along with the cone (254 mm diameter) supports the one-dimensional movement of the specimen. On top of the flange, the moving parts were mounted. A load cell fixed between the flange and the clamp of the central part measures the force applied on the specimen. Figure 7 illustrates the test setup with a specimen installed for simply supported bending type fatigue testing. While the specimen at the center was in surface contact (7 mm along the width and 3 mm along at length) with the central fixture, both ends maintained a line contact with bearings to ensure simply supported condition. In order to avoid any preloading on the specimen, spacers were used at both ends. The other two bearing holders were placed on top of the spacers. Finally, the heavy load toggle clamps fixed both ends of the test setup with the structure well. A high-speed laser-based displacement sensor pointing at the central clamp measured the displacement of the specimen. A proportional derivative (PD) controller was implemented to maintain the required set displacement while the load cell was used to measure the amplitude of the force applied. For further analysis, the displacement, load, and control signal amplitudes were recorded in a computer until the final failure of the specimen.

## 4. Results and Discussion

**Figure 8:**
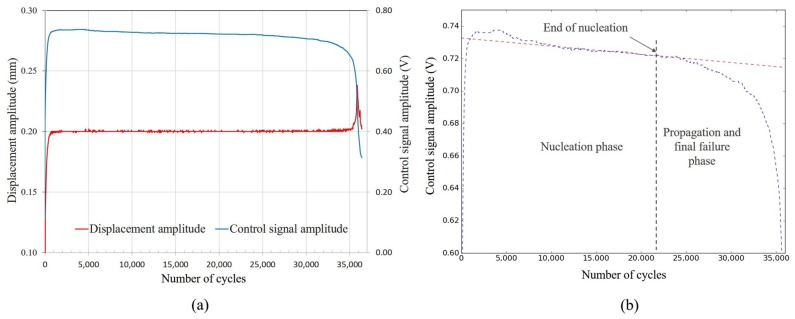
Controller response; (**a**): amplitude of the control signal and displacement for the horizontal specimen built with nominal parameter and (**b**): identification of the nucleation and propagation phase.

**Figure 9:**
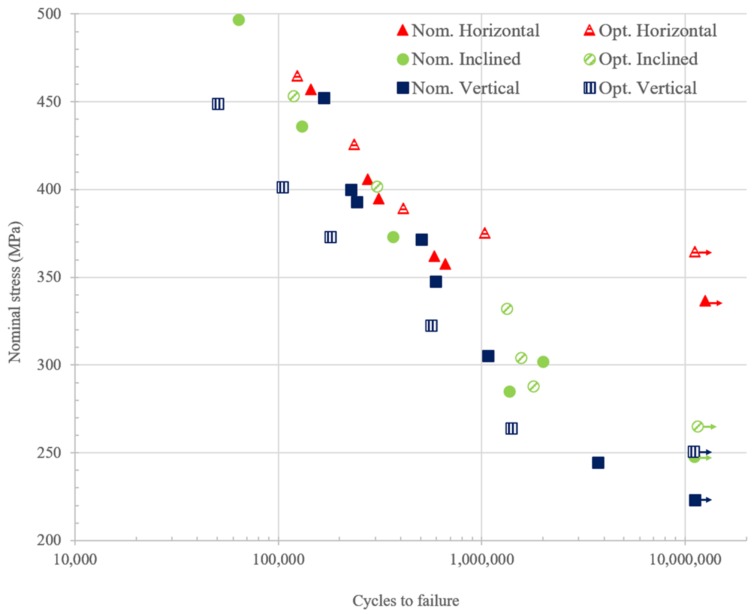
S-N plot of the specimens tested for horizontal, inclined, and vertical direction built with nominal and optimized parameters.

The fatigue test controlling the displacement was performed for simply-supported fully reversed (R = −1) type bending of the specimen with sinusoidal excitation at 56 Hz test frequency. All experiments were conducted at room temperature monitored with an infrared temperature sensor. The temperature variation reported here [51] remained within ±2 °C. The specimens failed at random locations within the gauges since defects are present randomly in the material. In Krouse type specimens, the stress concentration near the defects within the gauges was expected to be high, though the nominal stress distribution was assumed uniform. Figure 8 illustrates the controller performance in maintaining the set displacement of a horizontal specimen built with nominal parameters. During the test, the control signal amplitude was also monitored. In the fatigue test, with the crack initiation and propagation until the final failure happens, the stiffness of the specimen decreases. Therefore, the amplitude of the control signal also decreases for a constant displacement amplitude with an increasing number of cycles as shown in Figure 8b. The displacement increases suddenly at the final rapid failure stage indicating the complete failure of the specimen. While the stiffness of the test sample decreases at nucleation, propagation, and final failure stages, the rate of change in stiffness reduction is different at different stages. This gives us a unique comprehension of identifying nucleation and propagation phase applying a linear regression on the magnitude of the control signal and tracing the last peak of the signal near the line as ending of the nucleation phase. After identifying the nucleation and propagation, maximum nominal stress was calculated averaging the load amplitude up to the end of nucleation using Equation (3),

(3) $$\sigma =\frac{3F}{2kh^{2}},$$

where, *σ*, *F*, *h*, and *k* are the nominal stress, load amplitude, thickness of the specimen, and the slope of the wedge shape. Figure 9 shows the Wohler curve of the specimens tested for different build orientations fabricated with nominal and optimized parameters. As we can see, for both the nominal and optimized parameters, horizontal specimens demonstrate higher fatigue strength than the inclined and vertical specimens while inclined specimens have higher strength than vertical ones.

**Figure 10:**
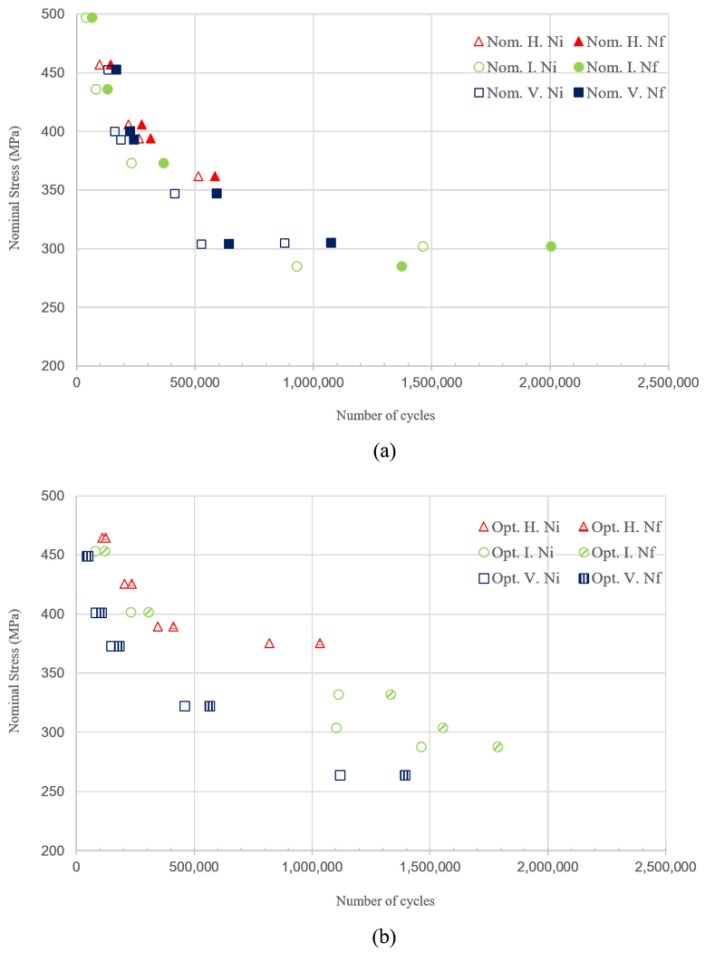
Nucleation phase and propagation and final failure phase of the specimens built with (**a**): nominal parameters and (**b**): optimized parameters. $N_{i}$ is the number of cycles at the end of the nucleation phase and $N_{f}$ is the number of cycles at the final failure of the specimen.

The fatigue strength of the material in the horizontal direction is higher not only in the nucleation phase but also during propagation as we can see from the analysis of the control signal [51] shown in Figure 10. During the fatigue test, the crack initiates within the gauge and propagates across the cross-section. In inclined and vertical specimens, the loading direction is parallel to the layers while this is normal for horizontal specimens. Moreover, inclined and vertical specimens consist of a larger number of layers hence interlayer within the gauges. In AM materials, interlayer strength is assumed to be weaker [8,31]. Additionally, the inclined and vertical specimens have a higher defect probability since they accommodate a larger number of interlayer within the gauges. Including anisotropy, these are the possible reasons for horizontal specimens demonstrating higher fatigue strength during nucleation and propagation. While comparing the inclined and vertical specimens, the crack in an inclined specimen has to travel through a large number of layers across the cross-section during propagation. Therefore the propagation cycle is longer in inclined specimens than in vertical specimens.

**Figure 11:**
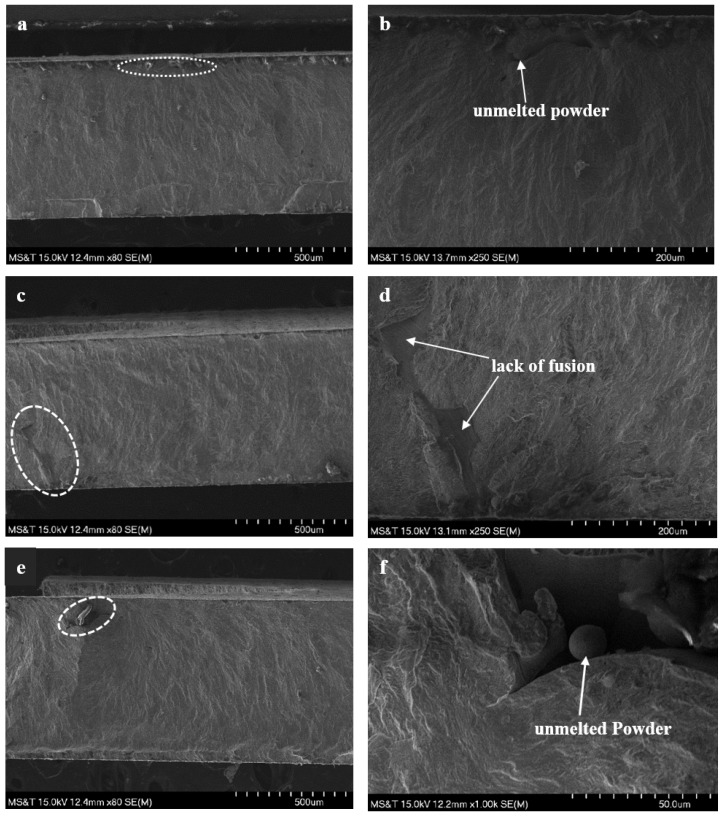
Scanning electron microscope (SEM) images of the fracture surfaces with possible crack initiation sites (marked by white dashed circles) of the specimens fabricated by the nominal parameters; (**a**): horizontal orientation; (**b**): A magnified view of crack initiation site in (a); (**c**): vertical orientation; (**d**): A magnified view of crack initiation site in (c); (**e**): inclined orientation; and (**f**): A magnified view of crack initiation site in (e).

**Figure 12:**
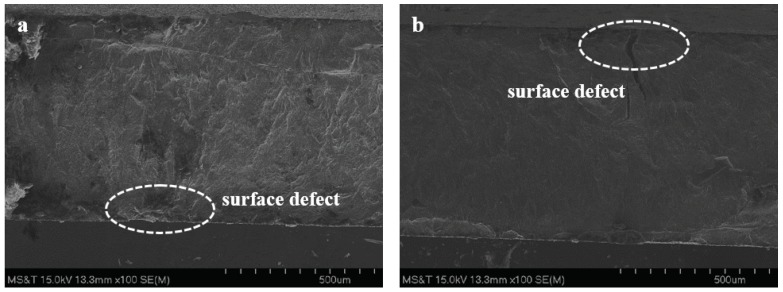
SEM images of the fracture surfaces with possible crack initiation sites (marked by white dashed circles) of the specimens fabricated by the optimized parameters; (**a**): horizontal orientation; (**b**): inclined orientation.

Scanning electron microscopic (SEM) images of fracture surfaces of the fatigue specimens fabricated with nominal and optimized parameters are shown in Figure 11 and Figure 12, respectively. Figure 11a,c,e exhibit the fracture surface of the horizontal, vertical, and inclined orientation specimens, respectively while the fracture surface for the horizontal and inclined specimens built with optimized parameters are shown in Figure 12a,b, respectively. In Figure 11b,d,f, nominal specimens with all orientations (horizontal, vertical, and incline) show that possible crack initiation sites are located around the lack-of-fusion defects close to the surface (circled by dashed lines). The initiation is a combined effect of higher nominal stress near the specimen surface and the stress concentration effect of defects. Especially in Figure 11b,f, unmelted powder particles can be found. While for the specimens built with optimized parameters, there is no obvious sign of lack-of-fusion type defects near the possible crack initiation sites, since the higher energy density provided for the optimized parameters can reduce the probability of the generation of lack-of-fusion. The initiation sites of specimens for optimized parameters mainly locate at the surface defect such as surface cracks (shown in Figure 12a,b). The fracture surface analysis reveals the possible reasons for the material fabricated with optimized parameters exhibiting higher fatigue strength at all different directions when compared with the material fabricated with nominal parameters.

## 5. Conclusions

In this paper, the effect of build process parameters on fatigue strength of laser powder bed fused AISI 304L stainless steel was investigated. Nominal and optimized process parameters were chosen to fabricate the material based on the density, tensile, and toughness test results. The fatigue test was performed for displacement control on a Krouse type miniature specimen with a dual gauge. The nucleation and propagation phase was identified implementing the control signal monitoring method. The analyses and experimental results show that material fabricated with optimized parameters demonstrate a higher fatigue strength at horizontal, inclined, and vertical directions than the specimens built with nominal parameters. Materials built with nominal parameters consist of the lack of fusion type defects mostly while the materials for the optimized parameters are expected to have keyhole type defects most. For particular stress, horizontal specimens have higher strength both during nucleation and propagation compared to the inclined and vertical specimens while the inclined specimens have higher strength than the vertical ones. For both the parameters, this is in good agreement with the anisotropy issue. Future studies may include the investigation of the size effect on fatigue performance, and the comparison of the fatigue behavior of LPBF 304L SS between as-built and annealed specimens.

## Figures and Tables

**Table 1:** Chemical composition of AISI 304L stainless steel powder particles in weight percentage.

Element	C	Cr	Cu	Fe	Mn	N	Ni	O	P	S	Si
wt.%	0.018	18.4	<0.1	bal.	1.4	0.06	9.8	0.02	0.012	0.005	0.6

**Table 2:** Process parameters used to fabricate 304L stainless steel.

Laser Power (W)	Exposure Time (μs)	Layer Thickness (μm)	Point Distance (μm)	Scan Speed (m/s)	Hatch Spacing (μm)	Energy Density (J/mm^3^)	ED #
			53	0.6	65	102.6	1
			53	0.6	75	88.8	2
			53	0.6	85	78.4	3
			53	0.6	95	70.2	4
			53	0.6	105	63.5	5
			70	0.8	65	76.9	6
			70	0.8	75	66.7	7
200	88	50	70	0.8	85	58.8	8
			70	0.8	95	52.6	9
			70	0.8	105	47.6	10
			88	1.0	65	61.5	11
			88	1.0	75	53.3	12
			88	1.0	85	47.1	13
			88	1.0	95	42.1	14
			88	1.0	105	38.1	15
